# Sinus of Valsalva Aneurysm: An Atypical Etiology of Recurrent Syncope

**DOI:** 10.7759/cureus.43325

**Published:** 2023-08-11

**Authors:** Muhammad Haseeb ul Rasool, Gowri Swaminathan, Asma U Hosna, Zara Bhutta, Allison Foster, Md Ripon Ahammed, Giovina Collura

**Affiliations:** 1 Medicine, Icahn School of Medicine at Mount Sinai / NYC Health + Hospitals Queens, New York, USA; 2 Otolaryngology - Head and Neck Surgery, KKR ENT Hospital & Research Institute, Chennai, IND; 3 Internal Medicine, Icahn School of Medicine at Mount Sinai / NYC Health + Hospitals Queens, New York, USA; 4 Cardiology, Icahn School of Medicine at Mount Sinai / NYC Health + Hospitals Queens, New York, USA

**Keywords:** syncope, arrhythmia, recurrent syncope, sinus of valsalva aneurysm, sinus of valsalva

## Abstract

The sinus of Valsalva presents the initial segment of the aorta from where the coronary vessels arise. Sinus of Valsalva aneurysms (SOVAs) present as progressive dilatation of the aortic sinus. SOVA arises both from the congenital and acquired weakness of the elastic lamina of the aortic media. Though most of the SOVAs are asymptomatic and diagnosed on screening for other pathologies, patients can present with symptoms of arrhythmia, aortic insufficiency, aorto-cardiac fistulas, and, in a few cases, with rupture. We describe a patient who presented with recurrent syncope and was found to have a 6 cm dilated SOVA with an ectatic ascending aorta. Further assessment revealed a left anterior fascicular block, aortic regurgitation, and mitral regurgitation. On further assessment, no other cause of syncope was found. There was no family history of aneurysm or sudden cardiac death. The patient was eventually discharged with outpatient follow-up with cardiothoracic surgery. In patients presenting with asymptomatic SOVA, a dilatation with a maximum diameter of 6.0 cm requires stringent monitoring and should be considered for surgery.

## Introduction

A sinus of Valsalva refers to the initially dilated segment of the ascending aorta, which extends between the aortic valve annulus and the sinotubular junction. The normal diameter of the sinus of Valsalva is less than 4.0 cm in men and 3.6 cm in women. The sinus of Valsalva functions to maintain blood supply to carotid arteries during systole when the aortic cusps open by preventing the occlusion of coronary artery ostia [1]. A sinus of Valsalva aneurysm (SOVA) presents abnormal dilatation of the aortic root, between the sinotubular junction and the aortic valve annulus. Histologically, the dilatation occurs due to weakness of the elastic lamina at the junction of the aortic media and annulus fibrosus [2].

SOVAs are approximately found in 0.09% of the general population based on autopsy findings, with SOVA accounting for 3.5% of the total congenital heart disease burden [3]. SOVA is classified as either congenital or acquired. Embryologically, the sinus of Valsalva forms as a blind diverticulum secondary to pressure forces on the aortic root. Aneurysmal dilatation of the sinus of Valsalva occurs due to connective tissue weakness from connective tissue disorders, such as Marfan’s syndrome and Ehlers-Danlos syndrome, and has been historically related to the presence of the bicuspid aortic valve [4]. Alternatively, acquired etiologies of SOVA have also been related to connective tissue and infectious diseases. Infectious etiologies that weaken the elastic tissue, resulting in acquired SOVA include syphilis, tuberculosis, and infective endocarditis. Connective tissue etiologies associated with SOVA include chronic atherosclerotic changes resulting in cystic medial necrosis, chest trauma, and iatrogenic injury during aortic valve replacement surgery [5]. Vasculitis involving the proximal aorta, such as Takayasu arteritis and inflammatory aortitis, can also lead to SOVA formation [6].

Non-ruptured SOVAs are usually asymptomatic and are found accidentally during other routine or screening imaging. Non-ruptured SOVA has been associated with significant aortic regurgitation in up to 50% of cases and has been associated with cardiac arrhythmias such as atrial fibrillation and complete heart block [5]. Occasionally, thrombus formation in SOVA leads to coronary ostial occlusion causing acute coronary syndrome. Contained rupture of right cusp SOVA can lead to fistula formation between the right atrium or right ventricle. It can also lead to a left to right fistula leading to right ventricular overload culminating in right heart failure [7].

We are describing a case of a patient presenting with recurrent syncope who was accidentally found to have an unruptured SOVA on further workup.

## Case presentation

A 68-year-old male with a prior history of right hip osteoarthritis and an active marijuana smoker presented after an episode of syncope. The patient suffered a syncopal episode while he was walking to the kitchen. He denied any pre-syncope chest pain, palpitation, headache, or dizziness. No seizure-like activity or pallor was noted by the family member. He also denied being confused, having headaches, or experiencing tinnitus after regaining consciousness. He affirmed having exertional shortness of breath and tiredness limiting his exercise tolerance over the last few months but denied any associated chest pain, orthopnea, or paroxysmal nocturnal dyspnea. He denied any significant past cardiac history and significant family history of premature cardiac death, congenital heart disease, or ruptured vascular aneurysms. He also denied any alcohol intake and cigarette smoking in the past and was a painter by profession.

**Figure 1:**
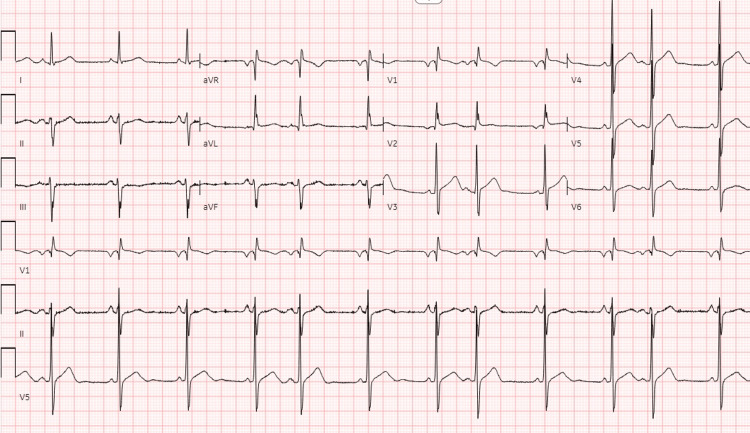
EKG revealing sinus rhythm at 72 beats per minute, with occasional premature atrial beats and left anterior fascicular block

Clinical exam at the time of admission was significant for age-appropriate blood pressure, and regular heart rate. Orthostatic vitals were negative for postural hypotension. The cardiac exam was significant for hyperdynamic circulation with an early diastolic murmur. The radial pulse was concurrent with a water-hammer pulse. An electrocardiogram revealed sinus rhythm at 72 beats per minute, with occasional premature atrial beats and left anterior fascicular block (Figure 1).

**Figure 2:**
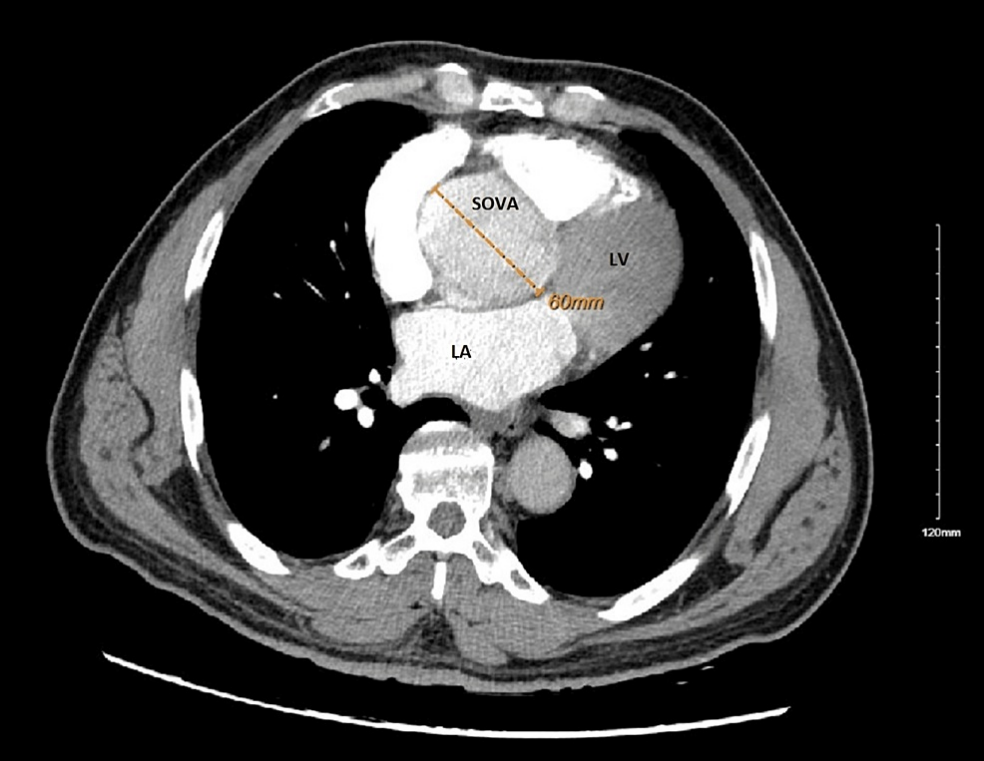
CT chest revealing 6 cm dilated sinus of Valsalva aneurysm LA: Left Atrium; LV: Left Ventricle; SOVA: SInus of Valsalva Aneurysm

**Figure 3:**
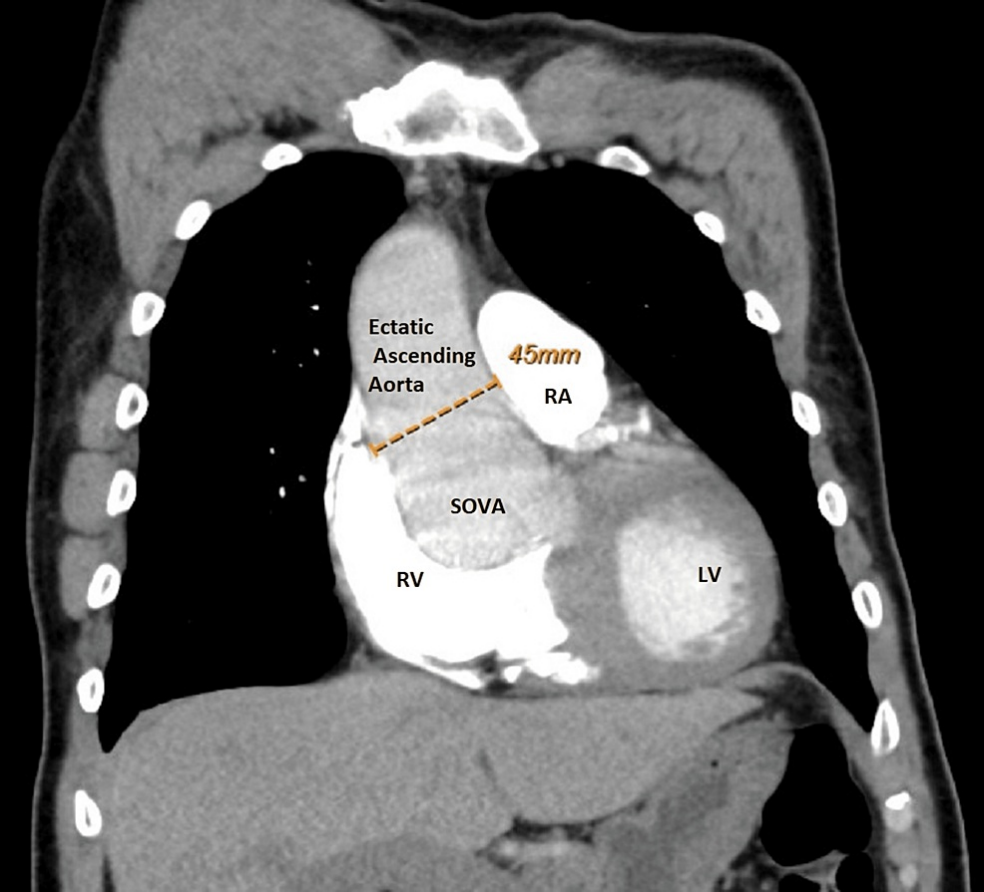
CT chest revealing ectatic ascending aorta to a diameter of 4.5 cm RA: Right Atrium; LV: Left Ventricle; RV: Right Ventricle; SOVA: SInus of Valsalva Aneurysm

**Figure 4:**
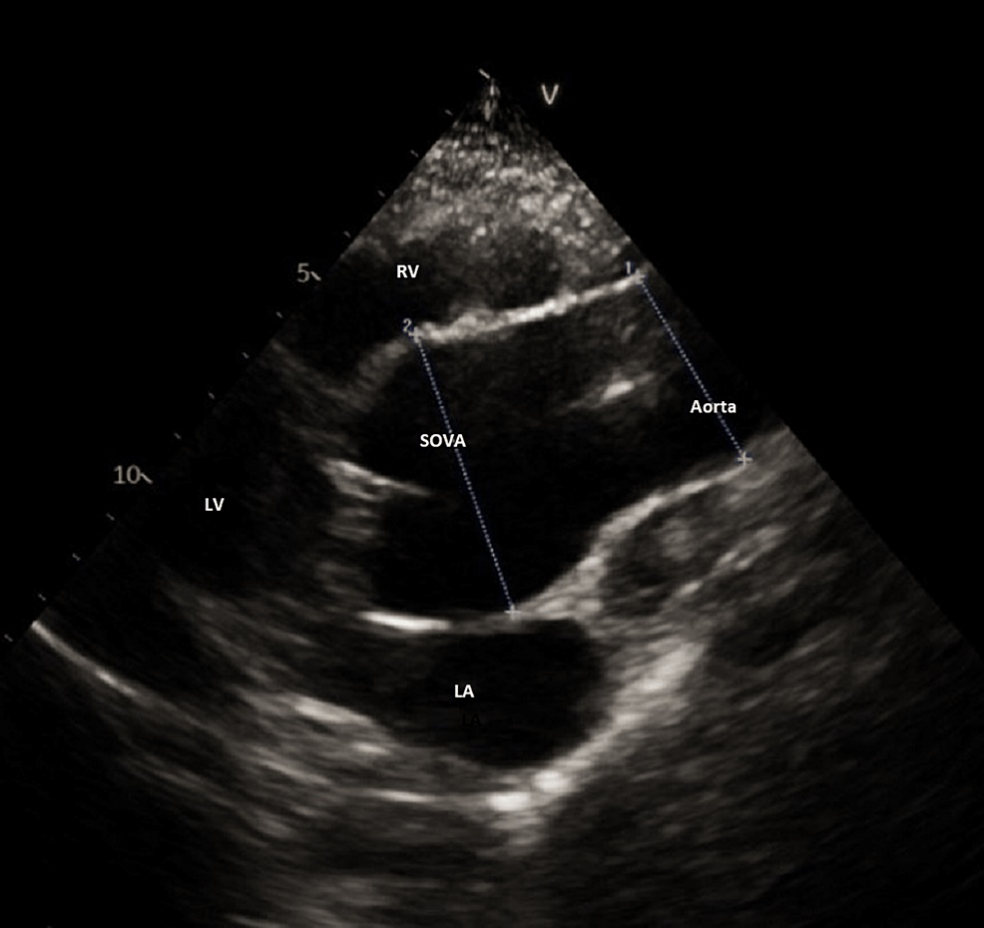
Echocardiogram revealing aneurysm of the sinus of Valsalva LA: Left Atrium; LV: Left Ventricle; RV: Right Ventricle; SOVA: SInus of Valsalva Aneurysm

**Figure 5:**
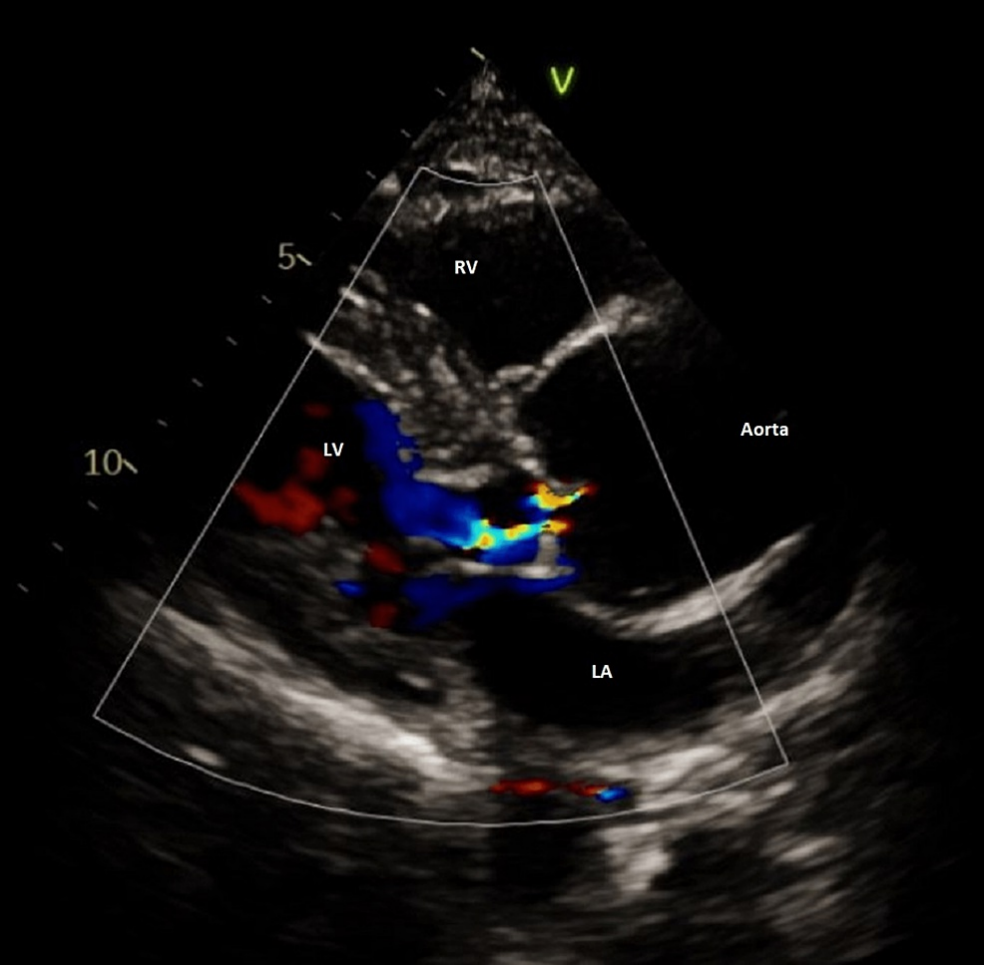
Echocardiogram revealing moderate aortic regurgitation and mitral regurgitation LA: Left Atrium; LV: Left Ventricle; RV: Right Ventricle

**Figure 6:**
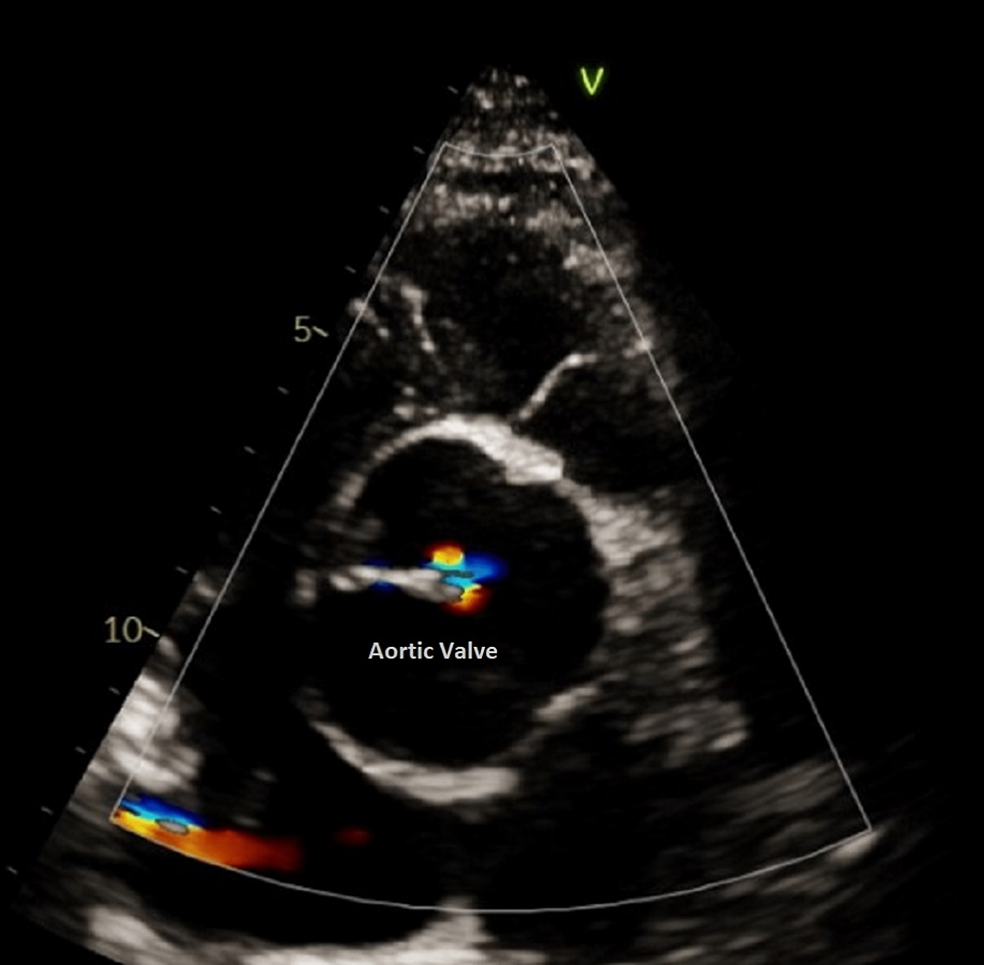
Echocardiogram revealing poor cooptation of the tricuspid aortic valve due to secondary aortic regurgitation

CT chest was performed with suspicion of acute pulmonary embolism, which revealed a 6 cm enlarged sinus of Valsalva and ectatic ascending aorta measuring 4.5 cm in maximum diameter, with no sign of thoracic aortic aneurysm or dissection. Aneurysm involved the right coronary and non-coronary cusp of the aorta (Figures 2-6).

Cardiovascular surgery was requested for assessment of the symptomatic SOVA and recommended that he should follow up in the outpatient clinic to continue further monitoring and intervene as necessary. He was placed on continued telemonitoring and did not show any malignant arrhythmia for the next 48 hours. The patient's syncope was attributed to possible transient conduction abnormality, as he already had a left anterior fascicular block and right bundle branch block (RBBB) on his EKG. He was eventually discharged with 30-day cardiac event monitoring and follow-up with the cardiology outpatient clinic. The cardiac event monitor assessment was unremarkable for any arrhythmia, and the patient is continuing to follow up with the cardiology clinic.

## Discussion

A SOVA is a discrete aneurysmal dilatation of one or more sinuses of Valsalva and is reported in only 0.09% of cases in a large autopsy series from 1914 [8,9]. Congenital defect of the sinus of Valsalva is an extremely rare cardiac anomaly, the true incidence of which is unknown. The 1914 autopsy series did not distinguish between congenital and acquired aneurysms [9]. The sinus of Valsalva is defined as the portion of the aortic root bounded by the aortic cusp, the aortic valve annulus, and the sinotubular ridge. Although the first reports of SOVA appeared as early as the nineteenth century [10], the first successful surgical repair using a cardiopulmonary bypass was recorded in 1957 by Lillehei et al. [11]. Usually originating from the right sinus or the anterior aspect of the noncoronary sinus of Valsalva, SOVA may present clinically as extracardiac or a free rupture into the pericardium [10]. Our 68-year-old male with a new diagnosis of unruptured SOVA is one of a kind and a value add to the existing literature on SOVA.

The morphology of SOVA can vary from a small, isolated enlargement of an aortic sinus to an extended finger-like or wind-sock-like appearance from the base or apex of a single aortic sinus [9,12]. Conditions like endocarditis, syphilis, Marfan’s syndrome, cystic medial necrosis, atherosclerosis, Loeys-Dietz syndrome, familial aortic dilatation and dissection, bicuspid aortic valve, Ehler-Danlos syndrome, and other connective tissue disorders [3,8,9]. There have been reports of SOVAs in individuals with a significant history of blunt chest trauma in the past [13]. In our patient, we ruled out infectious causes, any positive account of blunt chest trauma, and features suggestive of connective tissue disorders like Marfan’s and Ehler-Danlos syndrome. However, we could not exclude every possible etiology, as the patient had not had a noteworthy encounter before the one in question with any healthcare system in the United States or his home country, Jamaica.

SOVA is most frequently associated with the presence of a ventricular septal defect (VSD; 30% to 60%), aortic valve regurgitation (20%), bicuspid aortic valve (10%), pulmonic stenosis, coarctation of the aorta, atrial septal defect, and occasionally, coronary artery anomalies. Another known association is subvalvular aneurysms, where both the aortic and mitral annuli are afflicted. SOVAs are more common in Asian populations and have a notable male preponderance of 4:1 [12].

Patients with SOVAs may be completely asymptomatic or present with non-specific symptoms like dyspnea, chest pain, palpitations, or loss of consciousness [14]. Physical examination of these patients may not yield any significant findings unless the aneurysm is large or has ruptured. The most classic finding on auscultation is a continuous sawing-like murmur that occurs over both heart sounds; patients with aortic regurgitation may also present with a decrescendo diastolic murmur [14]. Our patient presented with sporadic syncopal episodes over the year before presentation to the hospital. His physical examination revealed a hyperdynamic circulation with an early diastolic murmur and positive water hammer pulse suggestive of aortic regurgitation.

The most dreaded clinical consequence of a SOVA that has been commonly reported across the literature is rupture. Ruptures typically occur between 20 and 40 years of age, with notable outliers in infancy or adulthood [15]. Rupture of the right and noncoronary sinuses typically results in communication between the aorta and the right ventricular outflow tract or the aorta or the right atrium, which is always clinically significant. Left SOVA rupture is clinically less significant, which usually results in communication between the left atrium and the left ventricular outflow tract [3,16]. In addition to the size of the aneurysm, and its location, with the right atrium being most common, the speed at which a rupture occurs is a significant determinant of clinical outcome in SOVA [3]. Symptoms of rupture can include substernal chest pain, abdominal pain, and mild to severe dyspnea; patients may also experience symptoms suggestive of acute heart failure, cardiac tamponade, hemodynamic compromise, and even sudden cardiac death [5]. In 1992, Elefteriades et al. did a prospective study on 1600 patients with thoracic aneurysms and dissection over 10 years and reported sharp “hinge points” in the aortic size at which rupture or dissection occurred. For the thoracic aorta, the hinge point is at 6 cm, and for the descending aorta, the hinge point is at 7 cm. By the time these sizes are reached, 31% of thoracic aortic aneurysms and 43% of descending aortic aneurysms have already ruptured [17]. Elefteriades et al. recommended that for a non-Marfan’s ascending aortic aneurysm, 5.5 cm should be the cutoff for surgical intervention, and for Marfan’s or familial ascending aortic aneurysm, it is 5 cm [17].

Cardiac computed tomography has gained popularity in SOVA imaging with useful contributing findings from traditional transthoracic echocardiography in case of a rupture. Other imaging modalities like MRI and contrast aortography are also being used to corroborate the clinical findings of a SOVA [3]. Cardiac CT and echocardiography, in our presented case, revealed a SOVA measuring 6 cm in diameter, arising from the right sinus and non-coronary sinus of Valsalva, along with ectatic ascending aorta measuring 4.5 cm in the largest diameter and moderate aortic regurgitation. We attribute the syncopal episodes in our patient to be probably from a possible mass effect from the aneurysm, obstructing the left ventricular outflow tract. Other potential factors could be moderate aortic regurgitation or conduction abnormalities from the left anterior fascicular block seen on the patient’s ECG.

Since the first successful surgical repair of SOVA by Lillhei et al., different approaches and repair techniques have been described in the literature. All the surgical repairs are performed with cardiopulmonary bypass and cardioplegic arrest. The choice of surgical approach depends on the presence or absence of aortic valvular pathology, the size of the SOVA, the presence of concomitant cardiac anomalies like VSD, and the cardiac chamber involved. The operative mortality rate for surgical management of SOVA is between 1.9% and 3.6%, with actual survival rates of close to 90% at 15 years [3], showing good long-term outcomes. For our patient, a collective decision was made by both the patient and the cardiothoracic surgical team that his condition would be closely monitored on an outpatient basis and that any new development would be managed appropriately.

## Conclusions

Our 68-year-old patient with a new diagnosis of SOVA, primarily asymptomatic until one year back, endorsing infrequent syncopal episodes only later in his sixth decade of life, is an outlier among the cases we reviewed for writing this case report. This leads us to believe that healthy patients with SOVAs can go on to lead functionally normal lives until complications like rupture or other associated features intervene, for example, aortic regurgitation along with enlarging aneurysm, like in this case. The management of SOVAs includes close monitoring and surgical intervention when necessary.
